# Urocortin in Second Trimester Amniotic Fluid: Its Role as Predictor of Preterm Labor

**DOI:** 10.1155/2009/947981

**Published:** 2009-11-04

**Authors:** C. Iavazzo, K. Tassis, D. Gourgiotis, M. Boutsikou, S. Baka, D. Hassiakos, C. Vogiatzi, L. Florentin-Arar, A. Malamitsi-Puchner

**Affiliations:** ^1^2nd Department of Obstetrics and Gynecology, Aretaieion Hospital, University of Athens, Athens 11528, Greece; ^2^Fetal Medicine Unit, “LITO” Maternity Hospital, Athens 11524, Greece

## Abstract

*Backgound*. The existence of a “placental clock” which determines the duration of gestation has been previously proposed. It is related to placental CRH secretion and is active from an early phase in human pregnancy. Urocortin is a specific ligand for the corticotropin-releasing factor (CRF) receptor expressed by human trophoblast and fetal membranes. The purpose of this study was to evaluate whether urocortin concentrations in the early second trimester amniotic fluid might serve to predict preterm delivery. *Method*. The urocortin concentrations in early second trimester amniotic fluid were measured in 41 pregnancies with term delivery and in 41 pregnancies with preterm delivery by using an immunoradiometric assay. Conditional logistic regression analysis was used for statistical analysis. *Results*. Mean amniotic fluid urocortin concentrations in women with preterm labor were 1.55 ± 0.63 ng/mL while those in women with term labor were 1.6 ± 0.49 ng/mL (p: NS). No statistical significant results were found when comparing amniotic fluid urocortin concentrations in women with preterm premature rupture of membranes leading to preterm labor (*n* = 19) to women with term delivery without premature rupture of membranes. *Conclusion*. These results suggest that urocortin concentrations in the amniotic fluid of genetic amniocentesis are not predictive of preterm labor and birth.

## 1. Introduction

Preterm labor affecting 10%–15% of all pregnancies [1] is responsible for increased perinatal mortality and morbidity, particularly for cerebral palsy [2]. Although various obstetric, anatomic, or medical risk factors are associated with preterm labor, 50% of such cases are idiopathic. An inflammatory process was proposed by many authors. Thus, increased amniotic fluid levels of cytokines as IL-6, TNF-*α*, IL1-ra, IL-8, ADAM-8, and ITAC [3–5] were determined and were considered to serve as predictors of preterm labor. However, other investigators suggest the “placental clock” model in the prediction of labor onset [6]. Thus, it was proposed that fetal or maternal stress might play a significant role in initiating the cascade of intracellular signals leading to preterm labour [7].

Corticotropin releasing factor (CRF) family comprises a variety of neuropeptides, CRF, and urocortin 1–3 [8]. Vaughan et al. [9] were the first to identify urocortin—a 40-amino-acid peptide which exhibits a 45% homology to CRF [10]—in the rat midbrain [9]. Urocortin is produced in the syncytiotrophoblast, deciduas, and fetal membranes [11–13]. Furthermore, Florio et al. stressed the important role of the fetus as a source of urocortin during labour [14].

Urocortins act on CRF receptors [15, 16]. More specifically, CRF-receptor-1 binds CRF and urocortin with similar affinity, whereas CRF-receptor-2 binds urocortin with a 40-fold higher affinity than CRF [16–18]. Both these neuropeptides bind to the receptors and act by activating G-protein-coupled signal transduction and cAMP production [17, 18]. 

Urocortin is characterized by specific properties which are implicated in the initiation of labor. Previous studies described that urocortins could increase myometrial contractility in an autocrine and paracrine manner through CRF-receptors-2 [19–26]. They could act directly [22] or indirectly through prostaglandins, estradiol, or ACTH secretion [7, 11, 24, 27, 28] on the myometrial cells. Sirianni et al. also pointed on the role of urocortin in triggering adrenal secretion of steroids relevant to parturition [20]. Moreover, urocortin elevates MMP-9 levels which lead to matrix disorganization and rupture of membranes [26]. Also, they regulate placental blood flow through the NO/cGMP pathway [19, 23]. Furthermore, a role of urocortin in the regulation of fetal adrenal function is also proposed [25]. Taken into account the properties of urocortin, we hypothesized that increased levels of amniotic fluid urocortin might predict, even from an early stage of pregnancy, women prone to deliver at term. Therefore, we aimed to determine urocortin levels in amniotic fluid, acquired during genetic amniocentesis, in women who delivered preterm as compared to those delivering at term and its possible influence on preterm premature rupture of membranes. To our knowledge, this is the first study to examine this issue.

## 2. Method

A prospective cohort study was performed in collaboration of the Second Department of Obstetrics and Gynecology, University of Athens, Aretaieion Hospital, and the Department of Fetal Medicine of “LITO” Maternity Hospital, Athens, Greece, during the period September 2005–December 2006. The study population consisted of Greek women with singleton pregnancies who presented for genetic amniocentesis. Women with twin pregnancies and women with known history of uterine abnormalities, cone biopsy, significant vaginal bleeding, and fetal malformations were excluded from the study.

Preterm labor was defined as labor before 37 weeks of gestation with regular uterine contractions (at least two uterine contractions/10 minutes during 30 minutes) in combination with the characteristic cervical changes [29–32]. Preterm premature rupture of the fetal membranes was defined as the rupture of the amniotic membranes with release of the amniotic fluid more than one hour before the onset of preterm labor (before 37 weeks of gestation) [33]. Gestational age was calculated from the last menstruation and was confirmed during routine ultrasound in the second trimester (16–19 weeks of gestation). Microbial invasion of the amniotic fluid was defined in our study as positive PCR for Mycoplasma hominis and Chlamydia trachomatis and/or growth of any bacteria (aerobic or anaerobic) in the amniotic fluid cultures except for coagulase-negative Staphylococcus, which was considered to be a skin contamination. All patients were followed until delivery for the occurrence of pregnancy complications. An independent investigator kept the medical records and entered maternal and perinatal data into a database. The ethics committee of our teaching hospital approved the study. Each woman gave informed consent before enrolment in the study and completed a questionnaire including questions regarding personal data, personal history, and family history.

Ultrasound-guided transabdominal amniocentesis with a 21-gauge needle was performed under aseptic conditions in 362 women during genetic amniocentesis. Amniocentesis was performed for advanced maternal age and/or increased risk for aneuploidy during nuchal translucency ultrasound. The first 0.5 mL of amniotic fluid was discarded to avoid maternal contamination. Twenty mL of amniotic fluid was aspirated from each woman (15 mL were used for genetic diagnosis). One mL of the uncentrifuged amniotic fluid was then immediately transported to the microbiological laboratory and was cultured for aerobic and anaerobic bacteria. Another 1 mL of the uncentrifuged amniotic fluid was also tested by polymerase chain reaction-PCR for Mycoplasma hominis and/or Chlamydia trachomatis detection. The remaining 3 mL of amniotic fluid were immediately placed in a refridgerator (+4°C) and were centrifuged within the next 6 hours at 3000 g and +4°C for 10 minutes. The supernatant was stored in polypropylene tubes at −80°C until analysis in order to avoid urocortin degradation.

Amniotic fluid urocortin levels were determined with an enzyme-linked immunosorbent assay (ELISA) (Urocortin (human), EIA Phoenix Pharmaceuticals INC (Burlingame, California, 94010)) which could measure urocortin in all biological fluids as long as its concentrations are above the kits detection limit. The amniotic fluid samples ran in duplicates. The intra- and interassay coefficients of variation CV% were <5% and <14%, respectively. The detection limit was 0.2 ng/mL with a linear range 0.2–3.8 ng/mL. It should be mentioned that the urocortin (Human) EIA Kit (Catalog No. EK-019-14) is specific for human Urocortin (100% cross reactivity) and shows 0% cross reactivity with human CRF, urocortin 2 or 3, and other substances (cortistatin 14, MCH, LH-RH, NPY, or somatostatin-28) [34, 35]. Laboratory personnel was blinded to the clinical history of the involved women.

Women who gave a spontaneous preterm delivery were defined as cases (*N* = 41) while, for each case, a woman matched for age with normal pregnancy served as control (*N* = 41). Furthermore, subgroup analysis was conducted in order to examine any possible association of urocortin levels with the incidence of preterm delivery with premature rupture of membranes. Nineteen women with preterm labor and premature rupture of membranes were defined as cases, while for every case a woman matched for maternal age delivering at term served as control (*N*1 = 19). Cases and controls delivered either spontaneously or by caesarean section.

All data except for age, gestational age at delivery, and gestational age at amniocentesis followed normal distribution (Kolmogorov-Smirnov test). Independent samples *t*-test was applied to detect differences between groups where continuous variables were normally distributed (urocortin and birthweight). Otherwise, Mann–Whitney *U*-test was applied. Pearson's Chi square test was used to detect differences between categorical variables. Conditional logistic regression analysis was used to examine the possible associations of urocortin with preterm labor. STATA 8.2 and SPSS 11.5 edition were used for the analysis. A *P*-value of <.05 was considered to be statistically significant.

## 3. Results

Out of 362 pregnant women who were included in the study, 41 had preterm labor (incidence: 11.26%) and a subgroup of 19 women delivered preterm with premature rupture of membranes (incidence: 5.22%). Ten were excluded after amniocentesis in the presence of fetal chromosomal abnormalities (two with trisomy 18, two with trisomy 21, one with Turner syndrome, one with Klinefelter syndrome, and other four with less usual karyotypes). Eight women were lost to follow-up. Four infants were delivered by caesarean section before the onset of labor for maternal (severe preeclampsia) or fetal reasons (compromised fetal growth or umbilical Doppler flow abnormalities). Two women delivered within 30 days following amniocentesis and were excluded from the study, as their delivery was considered related to the procedure of amniocentesis [36–40].

The demographic data of the study population are presented in Tables 1 and 2. No statistical significant differences concerning mean maternal age, gestational age at amniotic fluid sampling, indication for amniocentesis, and incidence of nulliparity were shown between the groups. Amniotic fluid cultures for common bacteria were negative, while Mycoplasma hominis and Chlamydia were identified in 2/338 and 2/338 subjects, respectively. However, one of the two women with Mycoplasma and one of the two women with Chlamydia delivered preterm.

**Figure 1:**
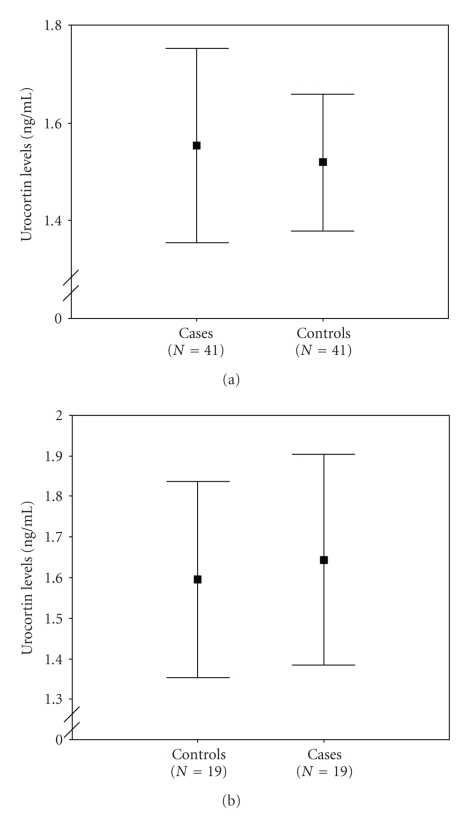
(a) Error bars of the concentrations of amniotic fluid urocortin from women with preterm delivery (cases) (Mean ± SD: 1.55 ± 0.63 ng/mL) and fullterm delivery (controls) (Mean ± SD:1.52 ± 0.43 ng/mL). Each box represents the mean (95% CI) concentration. (b) Error bars of the concentrations of amniotic fluid urocortin from women with preterm delivery and premature rupture of membranes (cases) (Mean ± SD:1.64 ± 0.54 ng/mL) and women with fullterm delivery (controls) (Mean ± SD: 1.6 ± 0.49 ng/mL). Each box represents the mean (95% CI) concentration.

Logistic regression analysis did not show any significant associations between urocortin levels and preterm delivery. Furthermore, no significant association between urocortin concentrations and the time interval between amniotic fluid sampling and delivery was observed. More specifically, the amniotic fluid levels of urocortin in women with preterm labor were not significantly higher than in women delivering at term (1.55 ± 0.63 ng/mL versus 1.52 ± 0.43 ng/mL) (Figure 1(a)). Moreover, no significant association was found between urocortin levels and preterm delivery with premature rupture of membranes. More specifically the amniotic fluid levels of urocortin in women with premature rupture of membranes were not higher than in women at term (1.64 ± 0.54 ng/mL versus 1.6 ± 0.49 ng/mL (Figure 1(b)).

## 4. Discussion

This is the first study which examined an association between amniotic fluid urocortin levels and possible prediction of preterm labor among asymptomatic women during the second trimester of pregnancy. The gene expression and localization of urocortin in syncytiotrophoblast, cytotrophoblast, and deciduas by using in situ hybridization and immunohistochemistry were reported in a study [11]. Also, other investigators showed that immunoreactive urocortin was detectable in maternal plasma from seven weeks of gestation and that such concentrations did not change as gestation progressed [41]. In another study, it was found that fetal plasma urocortin levels measured in umbilical cord artery and vein were increased in term and preterm labors [42]. Moreover, in the same study, it was revealed that maternal and fetal plasma urocortin levels increase at term or preterm vaginal labor compared to those after elective caesarean section [42]. It is known that the levels of CRF in maternal plasma increase throughout pregnancy, whereas urocortin remains constant [41, 43]. For this reason, many investigators proposed that urocortin might play an important role in human parturition and onset of labor [42, 44].

It has been stated that CRH and urocortin 1 appear to be predictors of the duration of gestation [27]. This could be explained by the fact that CRH through its receptors protects gestation by promoting myometrial quiescence via the generation of cAMP and cGMP and by upregulating nitric oxide synthase expression [14]. As proposed, urocortin has a significant role in uterine contractility in vitro by indirectly triggering a myometrial response through stimulation of placental adrenocorticotropin and prostaglandin release [7, 27]. Some investigators showed a distinctive role of CRF-receptors-2 in the control of uterine contractility during pregnancy [28]. Moreover, they suggested that urocortin 2 could have a dual role during pregnancy and labor: firstly, in the maintenance of myometrial relaxation and secondly, in the stimulation of contractility [28]. Other investigators trying to explain the possible mechanisms of myometrial stimulation found that CRH-induced activation is insufficient [21]. On the other hand, urocortin 1 could act through its receptors and could stimulate myometrial mitogen-activated protein kinase in cultured human pregnant myometrial cells [21].

In explaining preterm labor, the mechanism of the urocortin-activated placental, maternal, and fetal hypothalamo-pituitary-adrenal axis, as a response to stress, has been also implicated [20]. In this respect, urocortin has been used to test this theory. Thus, it has been proposed that ACTH, CRH, and urocortin 1 stimulate the fetal adrenal near term by increasing the production of cortisol and dehydroepiandrosterone sulfate and by activating CRF-receptor-1 [20]. The elevation in dehydroepiandrosterone sulfate levels could be used for placental estrogen synthesis, a fact that leads to parturition in humans [20]. At term, uterine contractility is proposed to be enhanced by upregulation of oxytocin receptor expression and communication between oxytocin and CRH receptors [21]. CRH/urocortins have a dual role: on the one hand by acting via their receptors and on the other by activating G-protein-coupled signal transduction and potentially by enhancing the oxytocin-driven generation of inositol triphosphate [19, 21–25]. Moreover, they generate—via their receptors—prostaglandins from the fetal membranes and deciduas; they play a role in placental vasodilation and they participate in fetal adrenal function and organ maturation in order to protect the fetus from environmental stress [19, 21–25]. Urocortin was also shown to induce a degradation of extracellular matrix by secretion of matrix-metalloproteinase-9 (MMP-9) with no changes in tissue inhibitors of MMP-1 (TIMP-1), a process leading to rupture of membranes [26].

Recent studies used maternal plasma urocortin between 28 and 34 weeks gestation to predict preterm delivery in women with threatened preterm labor [45]. They found that plasma urocortin was significantly higher in women who delivered preterm versus those delivering at term [45]. Based on the above findings, we investigated whether amniotic fluid urocortin levels during genetic amniocentesis could serve as a new marker predicting preterm labor. However, no statistically significant association was found neither in the group of preterm labor nor in the group of premature rupture of membranes group when compared with group of women delivering at term. In contrast to Florio et al. [45], we studied amniotic fluid urocortin levels at an earlier time point (second trimester) and in a different fluid (amniotic fluid versus maternal plasma). Furthermore, patients in the abovementioned study presented a threatened labor. It is noteworthy that our preterm delivery rate of 11.3 % occurred in a general and nonhigh-risk obstetric population who underwent amniocentesis for elevated maternal age or high nuchal translucency. Although our study did not find a predictive role of urocortin, the elevation of its levels with advancing gestation cannot be excluded. In conclusion, the present study did not find an association between urocortin levels in the second trimester and preterm delivery. Further studies are needed to elucidate the possible role of urocortin in human parturition.

## Figures and Tables

**Table 1:** Demographic data of women with preterm (*N* = 41) and term delivery (*N* = 41).

Groups	Preterm delivery (*N* = 41) Mean ± SD/Median (Range)	Controls (*N* = 41) Mean ± SD/Median (Range)	*P* value
Gestational age at delivery (weeks)	35.5 (22–37.6)	38.4 (37–40)	<.001
Gestational age at amniocentesis (weeks)	17.9 (15.1–25.4)	17.4 (15.9–23.7)	NS
Birth weight (g)	2469.7 ± 582.6	3172 ± 378	<.001
Age (years)	37 (27–43)	37 (27–44)	NS
<35 *N* (%)	15 (36.6)	13 (31.7)	—
>=35 *N* (%)	26 (63.4)	28 (68.3)	—
Parity	—	—	NS
First *N* (%)	16 (39)	15 (36.6)	—
Other *N* (%)	25 (61)	26 (63.4)	—
Smoking before pregnancy *N* (%)	16 (39)	15 (36.6)	NS
Smoking during pregnancy *N* (%)	8 (19.5)	9 (22)	NS
Alcohol Consumption before pregnancy *N* (%)	5 (12.2)	11 (26.8)	NS
Alcohol Consumption during pregnancy *N* (%)	1 (2.4)	3 (7.3)	NS
Use of drugs	13 (31.7)	18 (43.9)	NS

**Table 2:** Demographic data of women with preterm labor and premature rupture of membranes (*N* = 19) and women delivering at term (*N* = 19).

Groups	Preterm delivery with premature rupture of membranes (*N* = 19) Mean ± SD/Median (Range)	Controls (*N* = 19) Mean ± SD/Median (Range)	*P* value
Gestational age at delivery (weeks)	36 (22–37.6)	38.6 (37.3–39.9)	<.001
Gestational age at amniocentesis (weeks)	17.9 (15.6–25.4)	17.4 (15.9–23.7)	NS
Birth weight (g)	2508.3 ± 657.7	3234.2 ± 416.3	<.001
Age (years)	37 (27–43)	37 (29–44)	NS
<35 *N* (%)	5 (26.3)	6 (31.6)	—
>=35 *N* (%)	14 (73.7)	13 (68.4)	—
Parity	—	—	NS
First *N* (%)	9 (47.4)	7 (36.8)	—
Other *N* (%)	10 (52.6)	12 (63.2)	—
Smoking before pregnancy *N* (%)	7 (36.8)	6 (31.6)	NS
Smoking during pregnancy *N* (%)	4 (21.1)	2 (10.5)	NS
Alcohol Consumption before pregnancy *N* (%)	4 (21.1)	7 (36.8)	NS
Alcohol Consumption during pregnancy *N* (%)	1 (5.3)	1 (5.3)	NS
Use of drugs	4 (21.1)	7 (36.8)	NS
